# Investigating Clinical Failure of Bone Grafting through a Window at the Femoral Head Neck Junction Surgery for the Treatment of Osteonecrosis of the Femoral Head

**DOI:** 10.1371/journal.pone.0156903

**Published:** 2016-06-10

**Authors:** Wei Zuo, Wei Sun, Dingyan Zhao, Fuqiang Gao, Yangming Su, Zirong Li

**Affiliations:** 1 Department of Orthopaedic Surgery, China-Japan Friendship Medical School, Peking University Health Science Center, 38 Xueyuan Road, Haidian District, Beijing, 100191 China; 2 Centre for Osteonecrosis and Joint-preserving & Reconstruction, China- Japan Friendship Hospital, 2 Yingdong Road, Chaoyang District, Beijing, 100029 China; 3 Beijing University of Chinese Medicine, 2 Yingdong Road, Chaoyang District, Beijing, 100029 China; University of Umea, SWEDEN

## Abstract

**Aims:**

This study aimed to analyze the clinical factors related to the failure of bone grafting through a window at the femoral head-neck junction.

**Methods:**

In total, 119 patients (158 hips) underwent bone grafting for treatment of avascular necrosis of the femoral head. The patients were classified by their ARCO staging and CJFH classification. All patients were clinically and radiographically followed up every three months during the first year and every six months in the following year. The clinical follow-up comprised determination of pre- and postoperative Harris hip scores, while serial AP, frog lateral radiographs, and CT scan were used for the radiographic follow-up.

**Results:**

The clinical failure of bone grafting was observed in 40 patients. The clinical failure rates in patients belonging to ARCO stage II period, IIIa, and III (b + c) were 25.9%, 16.2%, and 61.5%, respectively, while those in patients belonging to (C + M + L1) type and L2, L3 type disease groups were 1.7%, 38.9%, and 39%, respectively. The clinical failure rates in patients aged below 40 and those aged 40 and over were 20.5% and 39.0%, respectively (all P < 0.05).

**Conclusion:**

Disease type, disease stage, and patient age are risk factors for failure of bone graft surgery. Patients belonging to ARCO stage II and IIIa showed a good overall response rate, while patients belonging to ARCO stage IIIb and IIIc and those with necrotic lesions involving the lateral pillar (L2 and L3 type) showed high surgical failure rates.

## Background

Osteonecrosis of the femoral head is a refractory bone disease. Previous reports have indicated that in the absence of appropriate therapy, the disease will progress in 1 to 5 years, eventually leading to femoral head collapse [1]. The collapse may be serious enough to affect joint function, leaving joint replacement surgery as the only viable treatment option. However, the long-term results of joint replacement surgery are unsatisfactory in young patients [2]. Effective hip-preserving surgery can delay the advancement of osteonecrosis, thus allowing a portion of the patients to retain their original joints and delay hip arthroplasty. The methods of hip-preserving surgery are as follows: core decompression, all sorts of osteotomies, as well as vascularized and non-vascularized bone graft. In 1994, Rosenwasser first performed the “lightbulb” surgery: opening a window at the femoral head-neck junction, debriding the necrotized tissue, and grafting bone tissue gathered from the patient himself/herself as well as artificial bone tissue. This contributes to the support of the femoral head and promotes bone tissue regrowth at the site of necrosis. Moreover, it does not affect the outcome of joint replacement surgery performed in the future [3,4,5,6,7]. Some patients showed good long-term clinical efficacy by this procedure. Experts have proposed that the approach of treating femoral head necrosis should be individualized based on patient age, and the etiology, stage, and type of the disease. However, the current reports on bone graft surgery are more focused on exploring its clinical efficacy, and there are few reports on the risk factors affecting postoperative clinical failure [5,6,7]. In this study, we retrospectively analyzed patients who underwent bone graft surgery in our hospital and discussed the clinical risk factors for failure of bone grafting through a window at the femoral head-neck junction.

## Materials and Methods

This study was approved by the Ethics Committee of China Japan Friendship Hospital. Written consents were provided by the patients to be stored in the hospital database and be used for clinical research.

This study belongs to the retrospective study. Our patients underwent operations from 2010 to 2013. We obtained the complete follow-up data of a total of 119 patients (158 hips). Prior to operation, all the patients were classified according to their MRI and CT scan results. Disease staging and the size of the necrotic lesions were assessed by the Association Research Circulation Osseous (ARCO) classification system. Disease type was classified according to the China-Japan Friendship Hospital (CJFH) classification system as M, C, L1, L2, and L3. According to the etiology, the disease was classified as corticosteroid-induced, alcohol-induced, and idiopathic. Among the 119 patients, 86 were male and 33 were female, with mean ages of 35.1 and 32.4 years, respectively. Inclusion criteria:1) Radiographic criteria of ARCO stage II-III. 2) Patient age below 55 years. 3) Informed consent for this study [6]. Exclusion criteria:1) Radiographic criteria of ARCO stage I and IV. 2) Patients due to other diseases need to continue to use glucocorticoid after operation [6]. We recorded down detailed personal information for all of our patients. After the patients’ operation, we strictly followed our follow-up guidelines and followed-up on all of our patients every three months during the first year after surgery and every six months in the following year, until the end of our research. During this time, we could not follow up on 8 patients (11 hips) during different periods of our follow-up due to the patients changing their contact information without notifying us. We ascertained the main endpoints by: The patient underwent hip joint replacement after his/her bone grafting operation, or the patient has a Harris score of <70 points.

### Surgical procedure

After anesthesia, the patient was laid in a lateral decubitus position. The Watson–Jones modified approach was followed, in which an approximately 5–7-cm incision was made over the greater trochanter for an anterolateral approach to the hip. The fascia lata was cut open in the direction of the skin incision, and the anterior gluteus medius was detached. The anterior joint capsule was cut longitudinally along the femoral head between the gluteus medius muscle and the tensor muscle of the fascia lata in order to expose the head-neck junction. A window to expose the bone (with a length and width of 1.5 cm and a depth of 0.5–1.0 cm) was made at the femoral head-neck junction. Under C-arm fluoroscopy, the necrotized bone located at the anterolateral and upper side of the femoral head was alternately debrided using a high-speed drill and a curette. The debrided area included the necrotized bone located at the weight-bearing surface, as well as partially sclerotic bones. The depth of the debridement reached the subchondral bone. For the sclerotic bone, multiple holes were created using a 2.5-mm drill until fresh blood oozed from the surface of the wound. Autologous cancellous bone, harvested from the iliac external circumferential lamella, was compressed layer by layer with the artificial bone (Shanghai Bio-lu Biomaterials Co. Ltd, Shanghai, China) into the debrided area. This procedure was completed under X-ray fluoroscopy to prevent the possibility of accidental bone cavities during the compression procedure. The bone window was covered with the originally excised bone plate and was fixed in place with an absorbable screw. After the surgery, patients were instructed to follow a strict rehabilitation and training program. Patients were allowed to leave the bed with crutches on the next day of the surgery. They were maintained at toe-touch weight bearing with crutches for 3 months. Higher impact loading activities were avoided in 12 postoperative months.

### Efficacy evaluation

The Harris hip score was used to evaluate the clinical efficacy of the operation. A score of less than 70 was classified as poor efficacy; a score between 70 and 80 was considered as decent efficacy; between 80 and 90, as good efficacy; and between 90 and 100, as superior efficacy. Serial AP and frog lateral radiographs were used for postoperative radiographic evaluation every three months during the first year after surgery and every six months in the following year. When necessary, CT and MRI scans were performed. The assessment of any change in the femoral morphology and determining the presence or absence of ossification in the bone grafting area were the primary objectives after the surgery. Clinical failure was defined as the need to perform total hip arthroplasty because of failure of operation, a Harris score of less than 70 points, and a progressive collapse of the femoral head (>2 mm compared to preoperative collapse) observed in postoperative scans.

### Statistical analysis

All statistical analyses were completed using the SPSS Statistical Software (SPSS for Windows, version 19.0). The means, standard deviations, and frequencies were calculated for general demographic and routine clinical data. We used the Cox risk model analysis, logistic regression analysis, and Kaplan-Meier survival curves for multivariate analysis. P values less than 0.05 were considered statistically significant.

## Results

**Fig 1:**
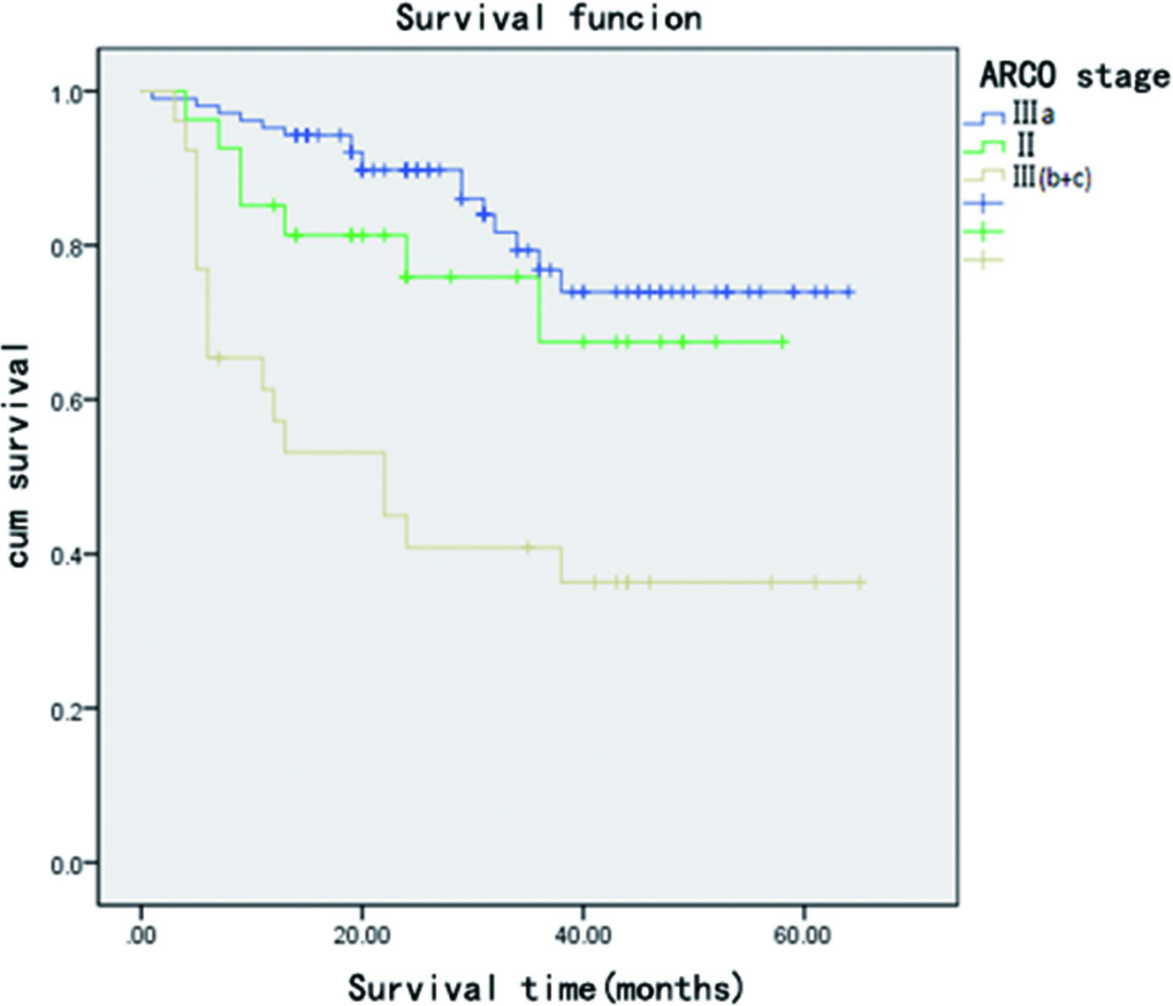
Kaplan–Meier survival curve shows that patients aged 40 and above had worse postoperative prognosis than patients aged under 40.

**Fig 2:**
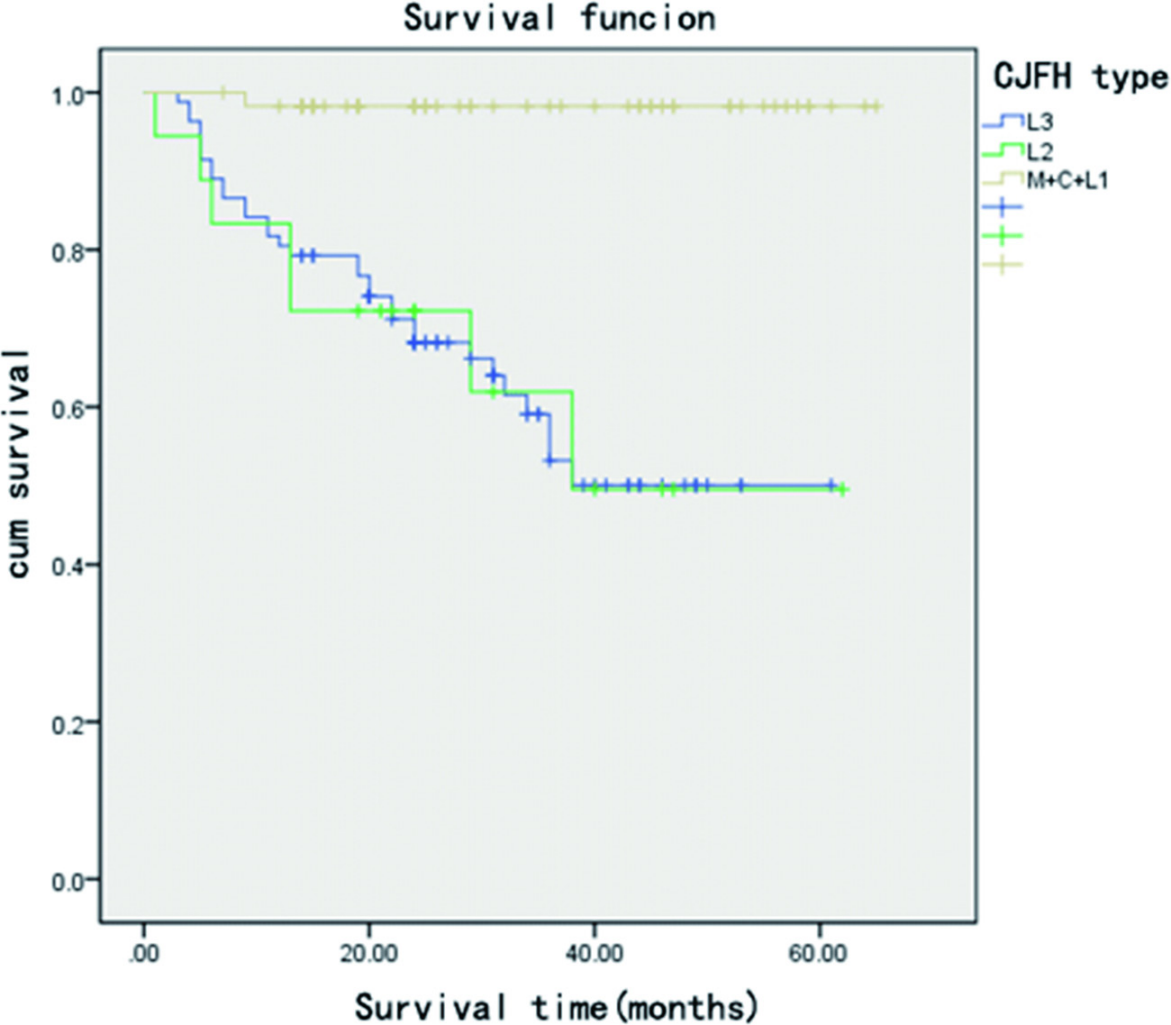
Radiographs of a 46-year-old patient with osteonecrosis of the femoral head of the right side. A) and B) Serial AP and frog lateral radiographs showing ONFH ARCO stage IIIa/CJFH type L2 on the right sides. Subchondral insufficiency fracture can be clearly observed on the frog lateral radiographs. C) and D) Preoperative magnetic resonance image shows that the necrotic lesions involve the lateral pillar. E) and F) Serial AP and frog lateral radiographs taken eight months post operation show a progressive collapse of the femoral head.

**Fig 3:**
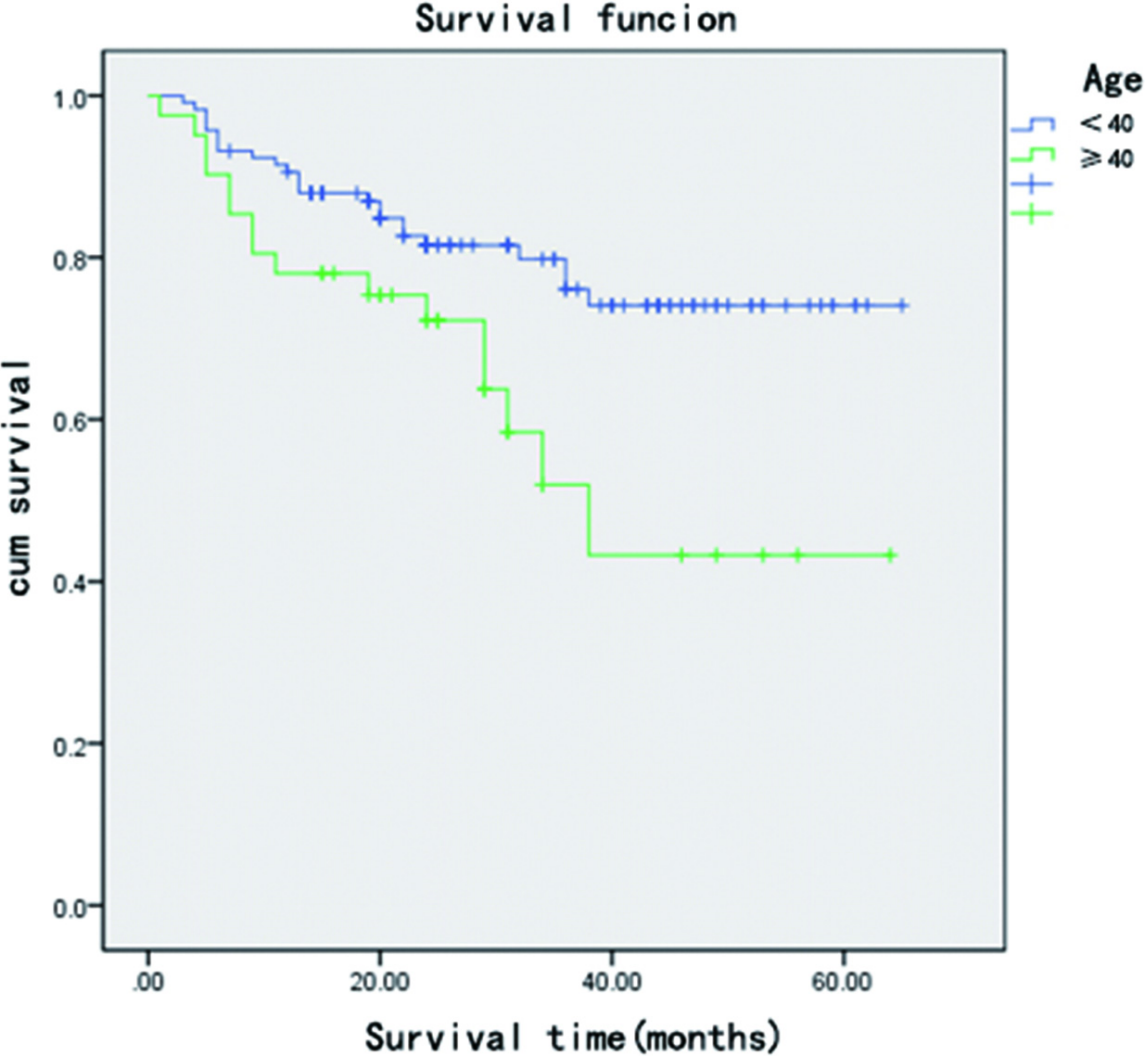
**Kaplan–Meier survival curve shows that hips with femoral head collapse degree >2 mm (ARCO stage IIIb, IIIc) are more prone to graft failure.** There were no significant statistical differences between ARCO stage IIIa and II.

**Fig 4:**
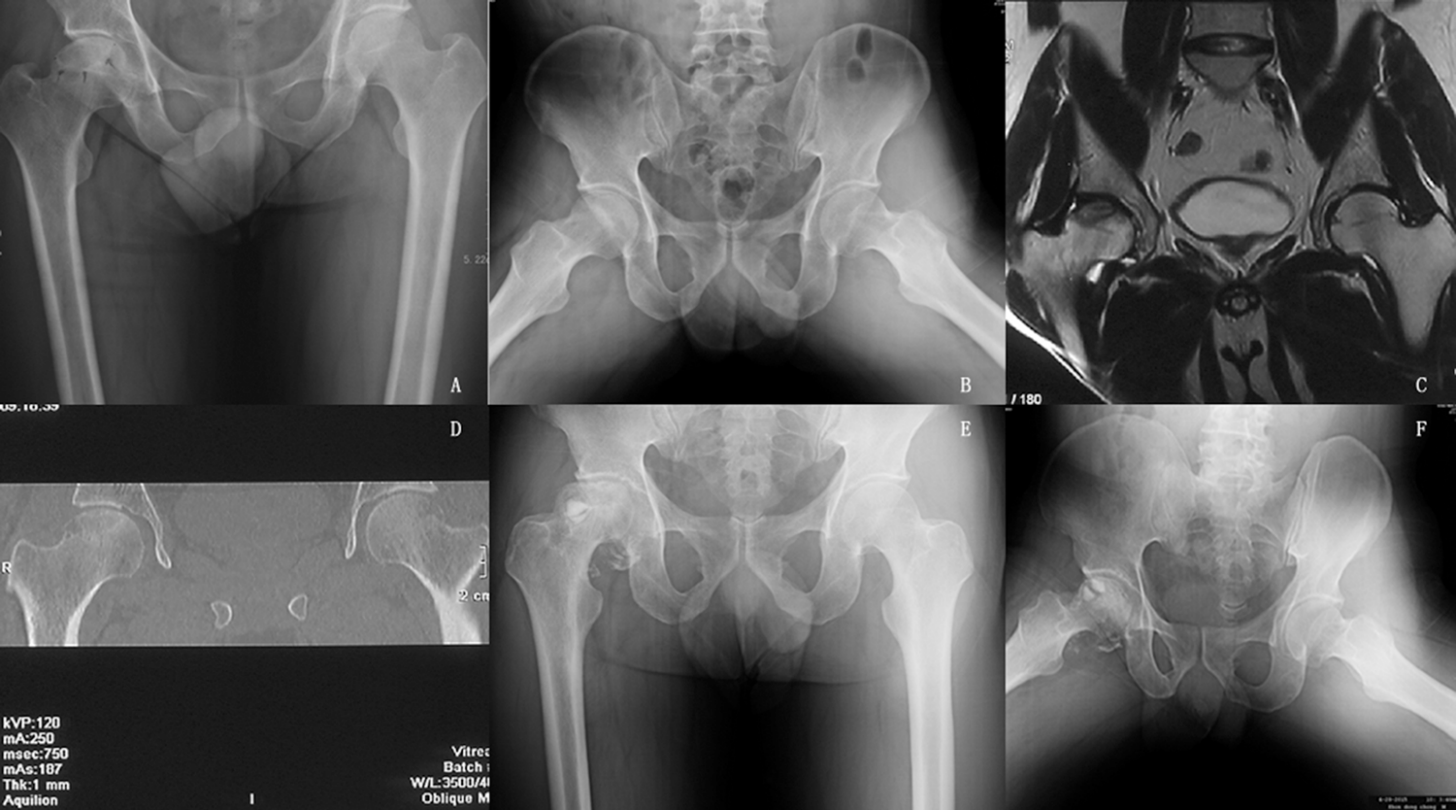
Kaplan–Meier survival curve shows that hips with necrotic lesions involving the lateral pillar (L2, L3 type) are more likely to fail bone grafting.

The average follow-up period for the 119 patients (158 hips) was 31.1 (range, 4–65) months. We followed up with all the patients that didn’t undergo hip arthroplasty for 2 years. Hip replacement surgery was required for 31 hips; 6 hips with a Harris score of less than 70 were subjected to serial AP and frog lateral radiography, which showed a progressive collapse of the femoral head (>2 mm compared to preoperative collapse). 3 hips with a Harris Score > = 70 were subjected to serial AP and frog lateral radiography, which showed a progressive collapse of the femoral head (>2 mm compared to preoperative collapse). These hips did not undergo total hip arthroplasty. The average post-operative score of all hips was 82.21(±13.62) points at the last follow-up or just before conversion to a total hip implant. Postoperative univariate analysis showed that disease type, disease stage, patient age, and preoperative Harris hip score are risk factors for failure of bone grafting through a window at the femoral head neck-junction (Table 1). The univariate analysis result of Harris hip score of different stages showed that Harris hip score is not a risk factor for clinical failure of different stages (all P>0.05) (Table 1). The Cox risk model showed that disease type, disease stage, and patient age are independent risk factors for postoperative clinical failure (Table 2). Patients aged 40 and above have a worse post-operational prognosis than patients aged below 40. (Table 2,Fig 1). The clinical failure rates for patients belonging to ARCO stage II, IIIa, and III (b + c) were 25.9%, 16.2%, and 61.5%, respectively, while those for patients belonging to (C + M + L1), L2, and L3 types were 1.7%, 38.9%, and 39%, respectively. The clinical failure rates for patients aged below 40 and those aged 40 and over were 20.5% and 39.0%, respectively (Table 1). The radiological failure rates for patients belonging to ARCO stage II, IIIa, and III (b + c) were 30.9%,35.2%, and 81.5%(Fig 2). The KM survival curve showed that the survival rate of patients belonging to ARCO stage III (b + c) was lower than that of patients belonging to ARCO stages IIIa and II. There were no significant differences in the survival rates of patients belonging to ARCO stages IIIa and II (Fig 3). The survival rates of patients with L2 and L3-type disease were lower than those of patients with (C + M + L1)-type disease. There were no significant differences in the survival rates of patients with L2 and L3 type disease (Fig 4).

**Table 1:** Patient summary.

	No. of patients (%)			
	Clinical success	Clinical failure	P-value	X^2^ (t)
Age (yrs)			0.018	5.503
≥40	25 (61.0)	16 (39.0)		
<40	93 (79.5)	24 (20.5)		
Gender			0.0793	0.069
Male	86 (74.1)	30 (25.9)		
Female	32 (76.2)	10 (23.8)		
Preoperative HHS			<0.001	18.261
0	9 (100.0)	0 (0.0)		
1	45 (86.5)	7 (13.5)		
2	49 (74.2)	17 (25.8)		
3	15 (48.4)	16 (51.6)		
Preoperative HHS of stage2			0.204	5.225
0	4(100.0)	0(0.0)		
1	9(81.8)	2(18.2)		
2	7(63.6)	4(36.4)		
3	0(0.0)	1(100.0)		
Preoperative HHS of stage3a			0.062	7.816
0	5(100.0)	0(0.0)		
1	35(92.1)	3(7.9)		
2	40(81.6)	9(18.4)		
3	8(61.5)	5(38.5)		
Preoperative HHS of stage3(b+c)			0.999	0.051
0	0(0.0)	0(0.0)		
1	1(33.3)	2(66.7)		
2	6(40.0)	9(60.0)		
3	3(37.5)	5(62.5)		
Preoperative stage			<0.001	22.673
ARCO stage II	20 (74.1)	7 (25.9)		
ARCO stage IIIa	88 (83.8)	17 (16.2)		
ARCO stage III (b + c)	10 (35.8)	16 (61.5)		
Preoperative type			<0.001	26.977
M + C + L1	57 (98.3)	1 (1.7)		
L2	11 (61.1)	7 (38.9)		
L3	50 (61.0)	32 (39.0)		
Etiology			0.384	3.048
Steroid-related	61 (71.8)	24 (28.2)		
Alcohol-related	18 (85.7)	3 (14.3)		
Idiopathic	39 (75.0)	13 (25.0)		
BMI	24.22 ± 3.897	23.730 ± 3.562	0.398	0.848 (t)

Harris score (0: 90–100; 1: 80–90; 2: 70–80; 3: <70); ARCO: Association Research Circulation Osseous; BMI: body mass index; HHS: Harris hip score.

**Table 2:** Multivariate cox regression analysis results.

Variable	B	Sb	Wald	p-value	RR
Preoperative Harris hip score	−0.226	0.225	1.014	0.314	0.798
CGFH type			8.968	0.011	
(M + C + L1) type	−2.921	1.023	8.159	0.004	0.054
L2 type	0.289	0.451	0.410	0.522	1.334
ARCO stage			15.855	0.000	
ARCO II	0.779	0.460	2.873	0.090	2.180
ARCO III (3b + 3c)	1.659	0.419	15.657	0.000	5.253
Age	1.044	0.358	8.499	0.004	2.841

Disease type, stage, and patient age were independent risk factors for postoperative clinical failure.

## Discussion

Osteonecrosis of femoral head is an intractable disease commonly observed in orthopedics. It often afflicts young and middle-aged individuals. The long-term efficacy of arthroplasty treatment for this condition is still unknown. Therefore, a treatment method that retains the joints of the affected patient is important. However, any kind of femoral head preserving surgical treatment cannot guarantee the same results for all patients. Postoperative joint function and improved clinical symptoms may not be achieved, and the femoral head may continue its progressive collapse, consequently requiring the patient to undergo joint replacement surgery again. This is inextricably linked with the individual differences among patients. Through retrospective research, we explored the factors affecting clinical failure rates of bone grafting through a window at the femoral head-neck junction, as discussed below.

### Disease stage

The effect of the disease stage on femoral head-saving surgery has always been a research hotspot. The patients in this study were classified according to their ARCO stages. Since no patient belonged to ARCO stage I, we divided the patients into IIa, IIb, IIc, IIIa, IIIb, and IIIc stages. This study discusses the correlation between post-operation treatment efficacy and the disease stages with regard to three aspects: the early precollapse stage (IIa, IIb, IIc) and postcollapse stage (IIIa, IIIb, IIIc), the area of necrosis (IIa, IIb, IIc), and the difference in the degree of collapse (IIIa, IIIb, IIIc), respectively, for bone grafting through a window at the femoral head-neck junction.

#### 1. Correlation between the presence or absence of femoral head collapse and clinical failure rate

Lieberman et al. systematically studied 48 relevant articles that analyzed the correlation between the existence of femoral head collapse and failure rates of hip-saving surgery, and observed that of the 2,163 pre-collapse (ARCOI, II) patients, 409 (19%) had to eventually undergo joint replacement surgery. Of the 1,463 patients with collapsed femoral head (ARCO stage III), 442 patients (30%) had to ultimately undergo joint replacement surgery [8]. However, some hip-saving surgeries, such as core decompression, are not suitable for patients with collapsed femoral heads, as shown by several reports [5,9]. Thus, the overall results may be affected. However, the results of various reports on bone graft surgery have also been mixed. Mont et al. [10] reported that bone graft surgery for patients with pre-collapse of the femoral head and post-collapse of the femoral head had surgical failure rates of 18.2% and 52.9%, respectively. Chang Y et al. [11] reported had surgical failure rates of 0% and 50% in pre-collapse and post-collapse stage patients, respectively, while Wang et al. [12] reported that pre-collapse and post-collapse stage patients had surgical failure rates of 25.4% and 38%, respectively. The pre-collapse and post-collapse stage patients in our study had surgical failure rates of 25.2% and 25.9% (P > 0.05), respectively. Further studies involving a larger sample size are required to confirm whether femoral head collapse affects failure rates of bone grafting surgery.

#### 2. Correlation between degree of femoral head collapse and surgical failure rate

The efficacy of hip-saving surgery in patients with collapsed femoral heads has always been controversial. The patients in our study were classified by their ARCO stages as IIIa, IIIb, and IIIc according to the extent of the collapse (<2 mm, 2–4 mm, >4 mm). Mont et al. reported that their criteria for performing bone graft surgery was a femoral head collapse of <2 mm.Chen et al.’s research into bone grafting surgery for patients with femoral head collapse showed that patients belonging to ARCO stage IIIa and IIIb had surgical failure rates of 69% and 100%, respectively. In addition, they pointed out that bone grafting technique is not applicable to patients with femoral head collapse. However, our patients belonging to ARCO stage IIIa and III (b + c) had surgical failure rates of 16.2% and 61.5%, respectively, and this difference was statistically significant. Therefore, we believe that ARCO stage IIIa patients who undergo bone grafting surgery can achieve good clinical treatment efficacy, while ARCO stage IIIb and IIIc patients have high surgical failure rates. Mont et al. also reported that bone grafting surgery is suitable for patients with non-collapsed femoral heads and those with femoral head collapse of <2 mm (ARCO stage IIIa).

#### 3. Correlation between the amount of necrosis tissue and clinical failure rate

Numerous studies have reported that the amount of the necrosis is an important factor affecting the efficacy of hip-saving surgery, and the larger the necrotic area, the higher the rate of surgical failure [13–19]. Landgraeber et al. [20] reported that the amount of preoperative necrosis correlates significantly with treatment failure. Patients who were treated with advanced core decompression with a preoperative necrotic volume of less than 2500 mm3 have a relatively low failure rate of 13% in comparison to patients with a larger preoperative necrotic volume. However, in this study, the failure rates in patients belonging to ARCO stage IIa, IIb, and IIc were 0%, 25%, and 23.5%, respectively, and the difference was not statistically significant. This could be attributed to the small sample size of patients belonging to ARCO stage II. We are currently studying this aspect for further verification.

### Disease type

Among the numerous factors affecting the failure rates of hip-saving surgery, the location of necrotic lesions has been the focus of previous reports and studies. The Japanese Investigation Committee (JIC) for osteonecrosis of the femoral head (ONFH) classifies the disease type of acetabulum weight-bearing area into three equal parts: the inner, middle, and outer parts. It also classifies femoral head necrosis according to its corresponding acetabulum weight-bearing area as A, B, C1, and C2 types. Classen et al. [21] adapted advanced core decompression surgery to treat osteonecrosis of the femoral head, and they reported that defect type C had a significantly higher rate of femoral head collapse than the smaller defects sizes A and B. Lieberman et al. [8] systematically reviewed 5 reports on the correlation between necrosis location and clinical failure rates. The results suggest that when the femoral head necrotic area did not exceed the corresponding inner weight-bearing area by 30%, only one out of 22 cases (4.5%) was a clinical failure, while when the femoral head necrosis area exceeded the corresponding inner weight-bearing area by 60%, 41 out of 91 cases (45%) were clinical failures. When the necrotic area exceeded the outer edge of the acetabulum, 26 out of 43 cases (60.5%) were clinical failures. This indicated that the surgical failure rates of hip-saving surgery are closely related to the location of necrosis. The disease types in all patients were classified according to the CJFH method. Based on the relationship of the necrotic area with the lateral column, we classified disease types as those in which the necrotic area does not involve the outer lateral column (C, M, L1 types) and those in which the necrosis is cumulative to the outer lateral column (L2 and L3 types). The results showed that patients with M + C + L1 type of disease had an overall surgical failure rate of 1.7%, those with L2 type disease had a failure rate of 38.9%, and those with L3 type disease had a failure rate of 39% (P < 0.05). The differences were statistically significant, indicating that for patients who require femoral head bone grafting surgery, whether the necrosis involves the outer lateral column is an important factor affecting the success rates and prognosis.

### Patient age, gender, etiology, BMI, and pre-surgical Harris hip score

Our data showed that age is an independent risk factor for postoperative clinical failure. Patients aged 40 and above have a worse post-operational prognosis than patients aged below 40. This might be attributed to the greater bone-repairing ability and faster ossification in younger patients. However, further studies are required to verify this speculation. During surgery, we discovered that after the removal of necrotic bones, cancellous bone bleeding occurred much more often in younger patients, indicating better blood circulation. We found no significant correlation between factors such as etiology, preoperative Harris hip score, BMI, and gender, and clinical failure rates.

## Conclusions

In summary, disease type, stage, and patient age are risk factors that impact surgical failure rates. Bone grafting through a window at the femoral head-neck junction performed in patients with no femoral head collapse or in those with a degree of collapse <2 mm (ARCO stage IIIa) showed good clinical success rate. Patients with a degree of femoral head collapse >2 mm (ARCO stage IIIb and IIIc) and those with necrotic lesions involving the lateral pillar (L2 and L3 type) had a high rate of surgical failure. Further, patients aged 40 and over had worse post-operation prognosis than patients aged below 40.

## Supporting Information

S1 Dataset Relevant data underlying the findings described in manuscript. (XLS)

S1 File Clinical ethics examination, and approval form of China-Japan Friendship Hospital Original. (PDF)

S1 Table STROBE Statement. (DOCX)
