# Correction: Investigating Clinical Failure of Bone Grafting through a Window at the Femoral Head Neck Junction Surgery for the Treatment of Osteonecrosis of the Femoral Head

**DOI:** 10.1371/journal.pone.0160163

**Published:** 2016-07-25

**Authors:** Wei Zuo, Wei Sun, Dingyan Zhao, Fuqiang Gao, Yangming Su, Zirong Li

The images for Figs 1 and 3 are incorrectly switched. The image that appears as Fig 1 should be Fig 3, and the image that appears as Fig 3 should be Fig 1. The images for Figs 2 and 4 are also incorrectly switched. The image that appears as Fig 2 should be Fig 4, and the image that appears as Fig 4 should be Fig 2. The figure captions appear in the correct order.

**Fig 1:**
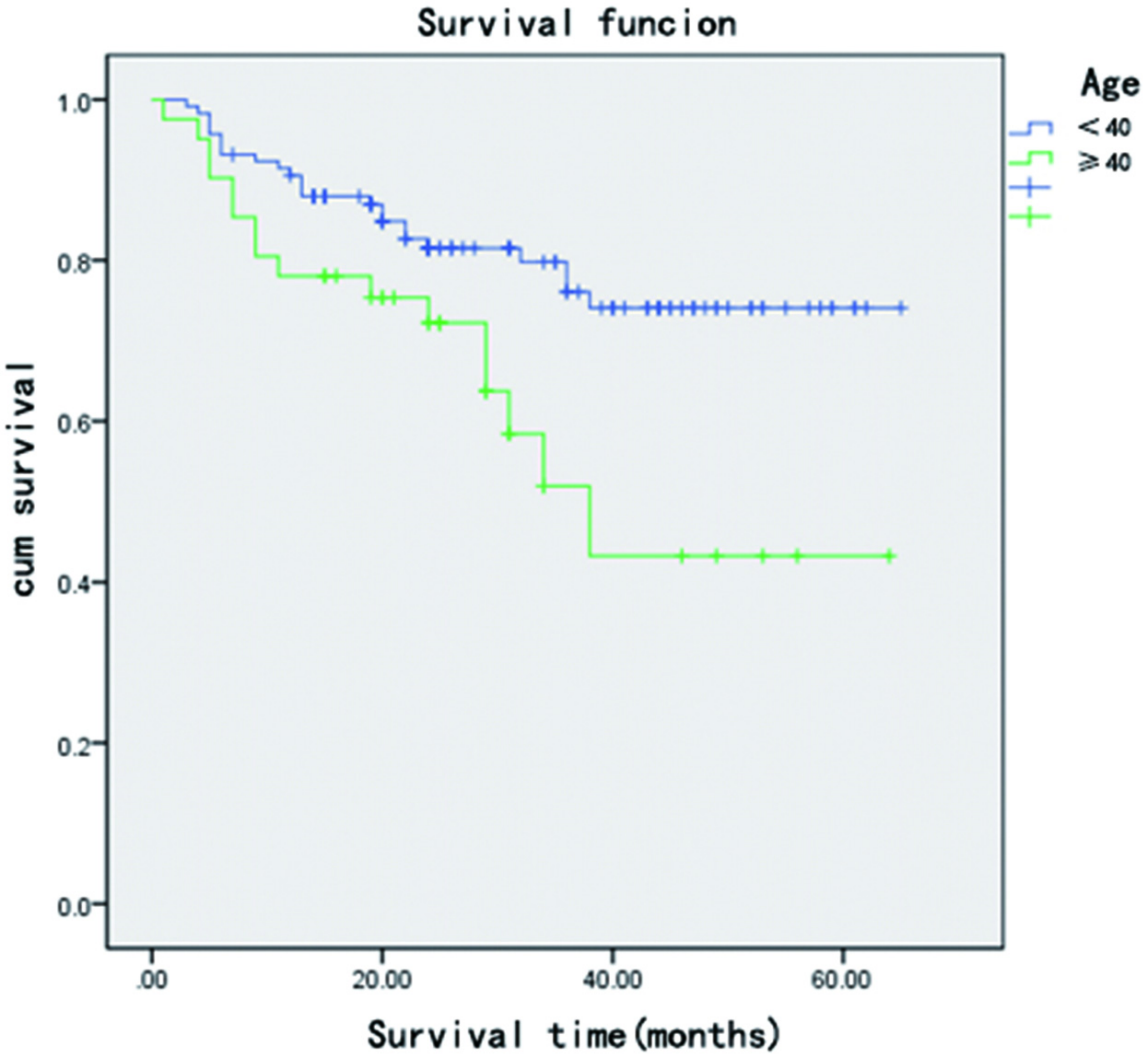
Kaplan–Meier survival curve shows that patients aged 40 and above had worse postoperative prognosis than patients aged under 40.

**Fig 2:**
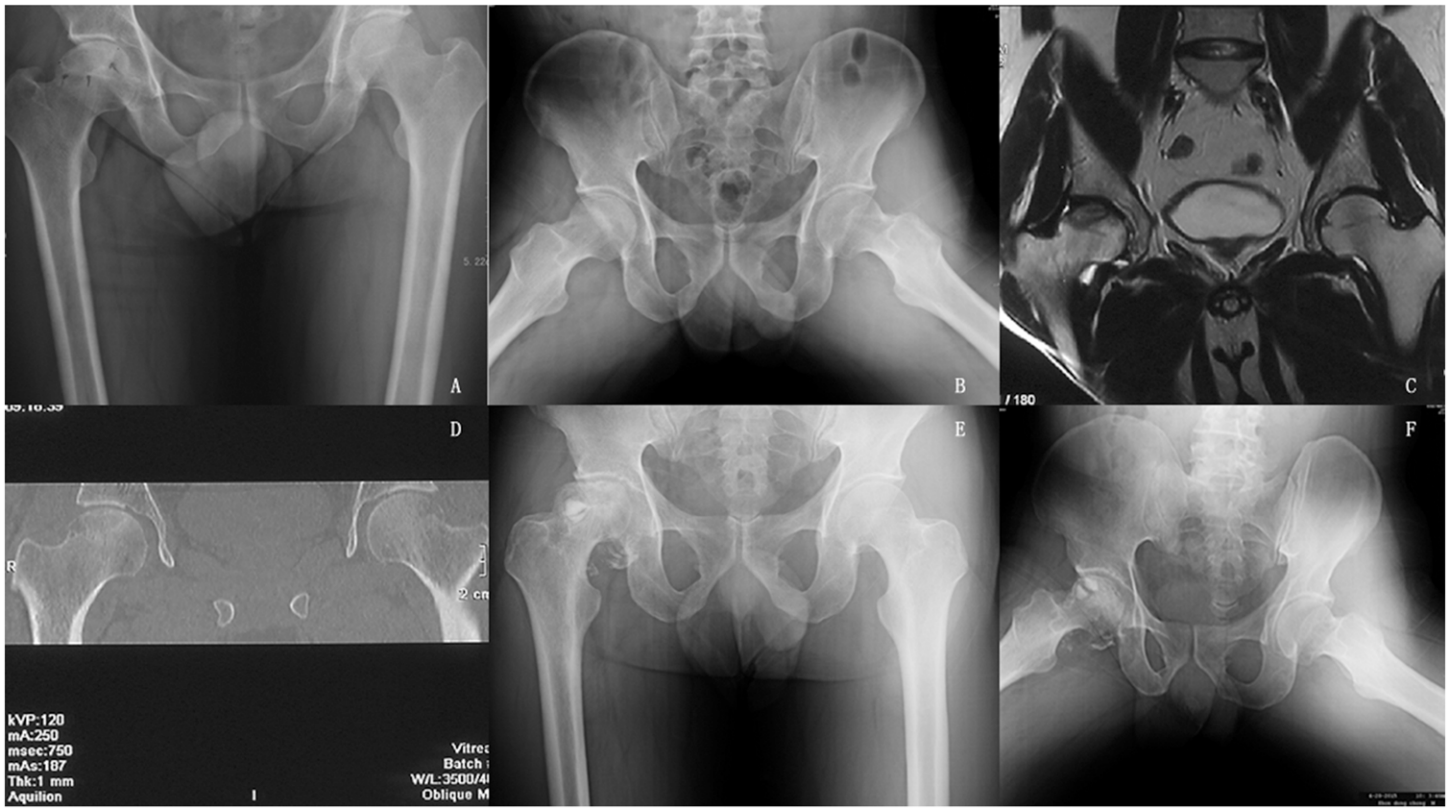
Radiographs of a 46-year-old patient with osteonecrosis of the femoral head of the right side. A) and B) Serial AP and frog lateral radiographs showing ONFH ARCO stage IIIa/CJFH type L2 on the right sides. Subchondral insufficiency fracture can be clearly observed on the frog lateral radiographs. C) and D) Preoperative magnetic resonance image shows that the necrotic lesions involve the lateral pillar. E) and F) Serial AP and frog lateral radiographs taken eight months post operation show a progressive collapse of the femoral head.

**Fig 3:**
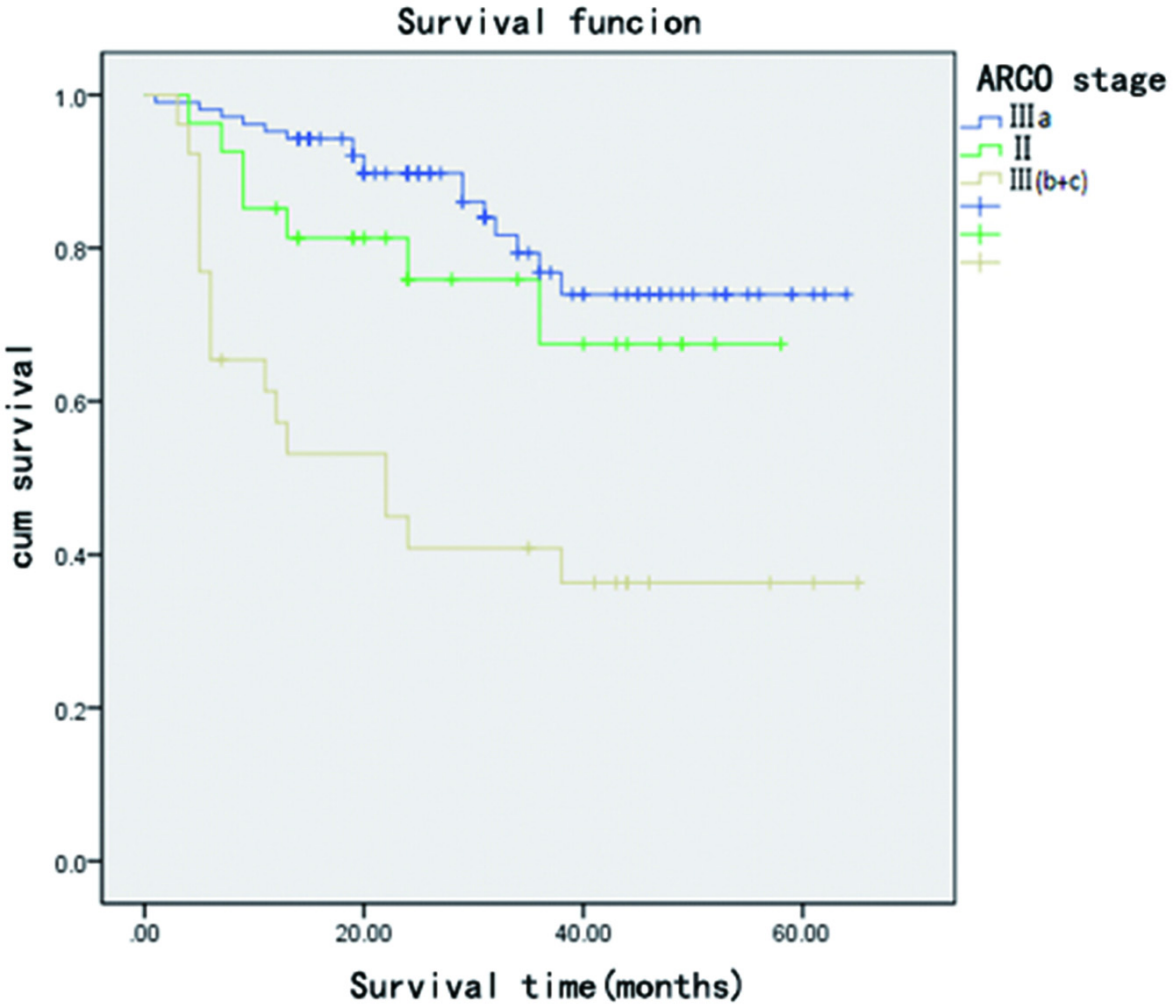
Kaplan–Meier survival curve shows that hips with femoral head collapse degree >2 mm (ARCO stage IIIb, IIIc) are more prone to graft failure. There were no significant statistical differences between ARCO stage IIIa and II.

**Fig 4:**
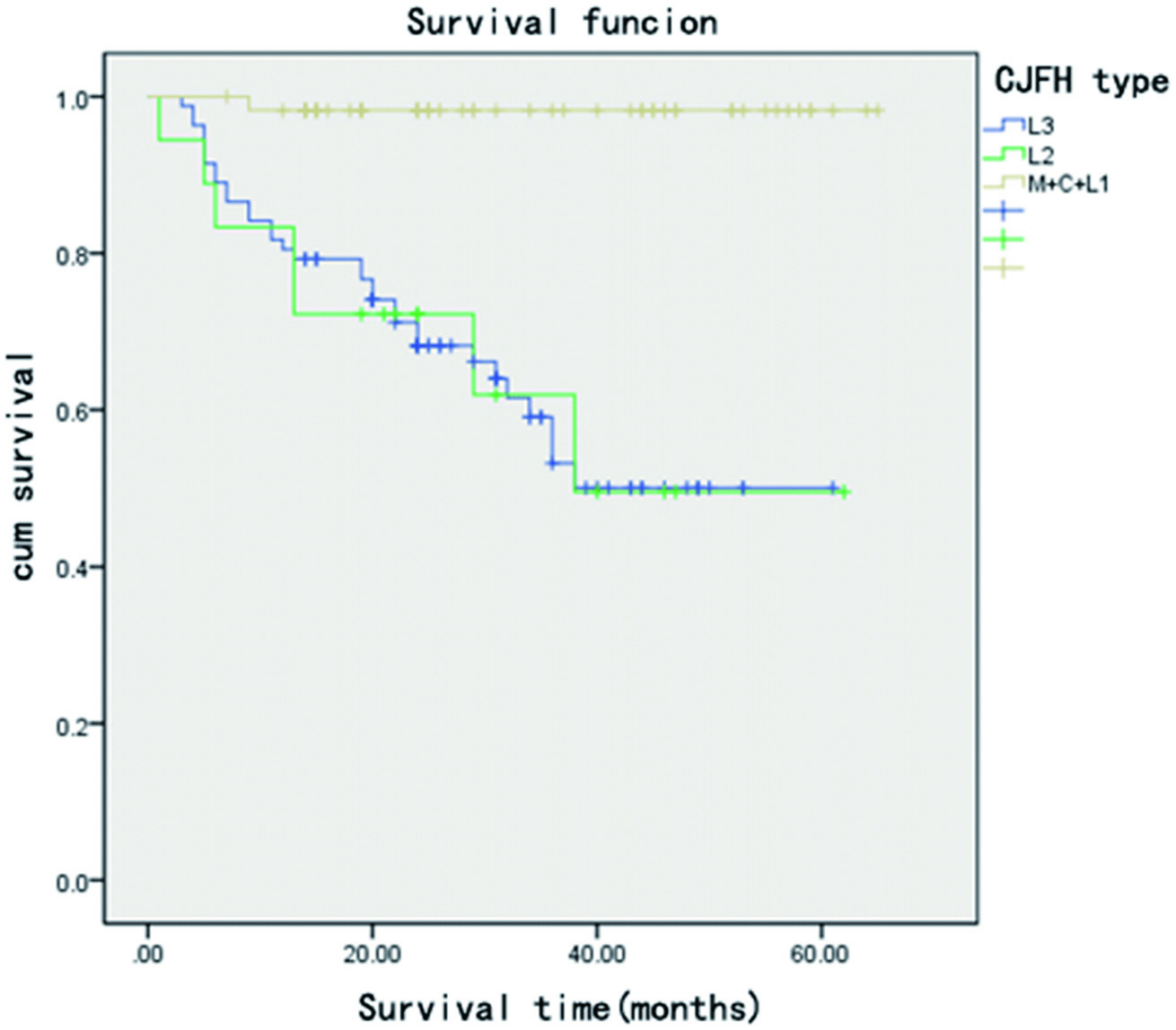
Kaplan–Meier survival curve shows that hips with necrotic lesions involving the lateral pillar (L2, L3 type) are more likely to fail bone grafting.
