# Supplementary material for: Investigating Clinical Failure of Bone Grafting through a Window at the Femoral Head Neck Junction Surgery for the Treatment of Osteonecrosis of the Femoral Head
Source: PLoS One. 2016 Jun 10;11(6):e0156903. doi: 10.1371/journal.pone.0156903 (PMC4902236; doi:10.1371/journal.pone.0156903)
Supplement: S1 File — (PDF) [file pone.0156903.s002.pdf]

中日友好医院临床伦理审批表

声明：本伦理委员会组成和工作程序符合 GCP 原则及国家相关法律法规

临床研究名称	常用技术对股骨头坏死保头治疗的中长期疗效分析及改进新技术探讨
项目类型	科研项目
申办单位	中日友好医院
科室	骨关节外科
项目负责人	李子荣 孙伟
审核材料	临床研究材料汇报
伦理审查意见	该临床研究目的明确，方案设计合理，符合生物医学研究伦理学的基本要求，经伦理委员会审查，同意该系列研究。<br>中日友好医院药物/器械临床试验伦理委员会<br>2015 年 10 月 22 日<br>中日友好医院 药品临床研究伦理委员会

地址： 北京市朝阳区樱花东街 2 号 邮编：100029 电话：010-84205708
Address：No.2 East Yinghua Road, Chaoyang District， Beijing 100029，China
Tel.Fax：86 10 84205708
