# Supplementary material for: Investigating Clinical Failure of Bone Grafting through a Window at the Femoral Head Neck Junction Surgery for the Treatment of Osteonecrosis of the Femoral Head
Source: PLoS One. 2016 Jun 10;11(6):e0156903. doi: 10.1371/journal.pone.0156903 (PMC4902236; doi:10.1371/journal.pone.0156903)
Supplement: S1 Table — (DOCX) [file pone.0156903.s003.docx]

STROBE Statement—checklist of items that should be included in reports of observational studies

|  | Item No. | Recommendation | Page  No. | Relevant text from manuscript |
| --- | --- | --- | --- | --- |
| **Title and abstract** | 1 | (*a*) Indicate the study’s design with a commonly used term in the title or the abstract | 2 | This study aimed to analyze the clinical factors related to the failure of bone grafting through a window at the femoral head-neck junction. |
|  |  | (*b*) Provide in the abstract an informative and balanced summary of what was done and what was found | 2 | Disease type, disease stage, and patient age are risk factors for failure of bone graft surgery. Patients belonging to ARCO stage II and IIIa showed a good overall response rate, while patients belonging to ARCO stage IIIb and IIIc and those with necrotic lesions involving the lateral pillar (L2 and L3 type) showed high surgical failure rates. |
| Introduction | | | |  |
| Background/rationale | 2 | Explain the scientific background and rationale for the investigation being reported | 3 | Some patients showed good long-term clinical efficacy by this procedure. Experts have proposed that the approach of treating femoral head necrosis should be individualized based on patient age, and the etiology, stage, and type of the disease. However, the current reports on bone graft surgery are more focused on exploring its clinical efficacy, and there are few reports on the risk factors affecting postoperative clinical failure. |
| Objectives | 3 | State specific objectives, including any prespecified hypotheses | 3 | In this study, we retrospectively analyzed patients who underwent bone graft surgery in our hospital and discussed the clinical risk factors for failure of bone grafting through a window at the femoral head-neck junction |
| Methods | | | |  |
| Study design | 4 | Present key elements of study design early in the paper |  |  |
| Setting | 5 | Describe the setting, locations, and relevant dates, including periods of recruitment, exposure, follow-up, and data collection | 3 | This study belongs to the retrospective study．Our patients underwent operations from 2010 to 2013. We obtained the complete follow-up data of a total of 119 patients (158 hips). After the patients’ operation, we strictly followed our follow-up guidelines and followed-up on all of our patients every three months during the first year after surgery and every six months in the following year, until the end of our research. During this time, we could not follow up on 8 patients (11 hips) during different periods of our follow-up due to the patients changing their contact information without notifying us. |
| Participants | 6 | (*a*) *Cohort study*—Give the eligibility criteria, and the sources and methods of selection of participants. Describe methods of follow-up  *Case-control study*—Give the eligibility criteria, and the sources and methods of case ascertainment and control selection. Give the rationale for the choice of cases and controls  *Cross-sectional study*—Give the eligibility criteria, and the sources and methods of selection of participants | 3 | Inclusion criteria:1) Radiographic criteria of ARCO stage II-III. 2) Patient age below 55 years. 3) Informed consent for this study. Exclusion criteria:1) Radiographic criteria of ARCO stage I and IV. 2) Patients due to other diseases need to continue to use glucocorticoid after operation. |
|  |  | (*b*) *Cohort study*—For matched studies, give matching criteria and number of exposed and unexposed  *Case-control study*—For matched studies, give matching criteria and the number of controls per case |  |  |
| Variables | 7 | Clearly define all outcomes, exposures, predictors, potential confounders, and effect modifiers. Give diagnostic criteria, if applicable | 3-4 | Prior to operation, all the patients were classified according to their MRI and CT scan results. Disease staging and the size of the necrotic lesions were assessed by the Association Research Circulation Osseous (ARCO) classification system. Disease type was classified according to the China-Japan Friendship Hospital (CJFH) classification system as M, C, L1, L2, and L3. According to the etiology, the disease was classified as corticosteroid-induced, alcohol-induced, and idiopathic. The Harris hip score was used to evaluate the clinical efficacy of the operation. Serial AP and frog lateral radiographs were used for postoperative radiographic evaluation every three months during the first year after surgery and every six months in the following year. When necessary, CT and MRI scans were performed. |
| Data sources/ measurement | 8* | For each variable of interest, give sources of data and details of methods of assessment (measurement). Describe comparability of assessment methods if there is more than one group | 3 | Prior to operation, all the patients were classified according to their MRI and CT scan results. Disease staging and the size of the necrotic lesions were assessed by the Association Research Circulation Osseous (ARCO) classification system. Disease type was classified according to the China-Japan Friendship Hospital (CJFH) classification system as M, C, L1, L2, and L3. According to the etiology, the disease was classified as corticosteroid-induced, alcohol-induced, and idiopathic. According to the etiology, the disease was classified as corticosteroid-induced, alcohol-induced, and idiopathic, Among the 119 patients, 86 were male and 33 were female, with mean ages of 35.1 and 32.4 years, respectively. |
| Bias | 9 | Describe any efforts to address potential sources of bias | 3-4 | Our patients underwent operations from 2010 to 2013. We obtained the complete follow-up data of a total of 119 patients (158 hips). We recorded down detailed personal information for all of our patients. After the patients’ operation, we strictly followed our follow-up guidelines and followed-up on all of our patients every three months during the first year after surgery and every six months in the following year, until the end of our research. During this time, we could not follow up on 8 patients (11 hips) during different periods of our follow-up due to the patients changing their contact information without notifying us. all the patients were classified according to their MRI and CT scan results. Disease staging and the size of the necrotic lesions were assessed by the Association Research Circulation Osseous (ARCO) classification system. Disease type was classified according to the China-Japan Friendship Hospital (CJFH) classification system as M, C, L1, L2, and L3. |
| Study size | 10 | Explain how the study size was arrived at | 3 | Our patients underwent operations from 2010 to 2013. We obtained the complete follow-up data of a total of 119 patients (158 hips). |

Continued on next page

| Quantitative variables | 11 | Explain how quantitative variables were handled in the analyses. If applicable, describe which groupings were chosen and why | 4 |  |
| --- | --- | --- | --- | --- |
| Statistical methods | 12 | (*a*) Describe all statistical methods, including those used to control for confounding | 4 | All statistical analyses were completed using the SPSS Statistical Software (SPSS for Windows, version 19.0). The means, standard deviations, and frequencies were calculated for general demographic and routine clinical data. We used the Cox risk model analysis, logistic regression analysis, and Kaplan-Meier survival curves for multivariate analysis. P values less than 0.05 were considered statistically significant. |
|  |  | (*b*) Describe any methods used to examine subgroups and interactions | 4 |  |
|  |  | (*c*) Explain how missing data were addressed | 4 |  |
|  |  | (*d*) *Cohort study*—If applicable, explain how loss to follow-up was addressed  *Case-control study*—If applicable, explain how matching of cases and controls was addressed  *Cross-sectional study*—If applicable, describe analytical methods taking account of sampling strategy | 4 |  |
|  |  | (*e*) Describe any sensitivity analyses | 4 |  |
| Results | | | | |
| Participants | 13* | (a) Report numbers of individuals at each stage of study—eg numbers potentially eligible, examined for eligibility, confirmed eligible, included in the study, completing follow-up, and analysed | 3 | Our patients underwent operations from 2010 to 2013. We obtained the complete follow-up data of a total of 119 patients (158 hips). During this time, we could not follow up on 8 patients (11 hips) during different periods of our follow-up. |
|  |  | (b) Give reasons for non-participation at each stage |  | we could not follow up on 8 patients (11 hips) during different periods of our follow-up. due to the patients changing their contact information without notifying us. |
|  |  | (c) Consider use of a flow diagram |  |  |
| Descriptive data | 14* | (a) Give characteristics of study participants (eg demographic, clinical, social) and information on exposures and potential confounders |  | table 2 |
|  |  | (b) Indicate number of participants with missing data for each variable of interest | 4 | During this time, we could not follow up on 8 patients (11 hips) during different periods of our follow-up. |
|  |  | (c) *Cohort study*—Summarise follow-up time (eg, average and total amount) | 4-5 | The average follow-up period for the 119 patients (158 hips) was 31.1 (range, 4–65) months. We followed up with all the patients that didn’t undergo hip arthroplasty for 2 years. |
| Outcome data | 15* | *Cohort study*—Report numbers of outcome events or summary measures over time |  | Hip replacement surgery was required for 31 hips; 6 hips were subjected to serial AP and frog lateral radiography, which showed a progressive collapse of the femoral head (>2 mm compared to preoperative collapse) with a Harris score of less than 70. 3 hips were subjected to serial AP and frog lateral radiography, which showed a progressive collapse of the femoral head (>2 mm compared to preoperative collapse) with a 70 or higher Harris score. |
|  |  | *Case-control study—*Report numbers in each exposure category, or summary measures of exposure |  |  |
|  |  | *Cross-sectional study—*Report numbers of outcome events or summary measures |  |  |
| Main results | 16 | (*a*) Give unadjusted estimates and, if applicable, confounder-adjusted estimates and their precision (eg, 95% confidence interval). Make clear which confounders were adjusted for and why they were included |  | Postoperative univariate analysis showed that disease type, disease stage, patient age, and preoperative Harris hip score are risk factors for failure of bone grafting through a window at the femoral head neck-junction. The Cox risk model showed that disease type, disease stage, and patient age are independent risk factors for postoperative clinical failure (Table 1). The clinical failure rates for patients belonging to ARCO stage II, IIIa, and III (b + c) were 25.9%, 16.2%, and 61.5%, respectively, while those for patients belonging to (C + M + L1), L2, and L3 types were 1.7%, 38.9%, and 39%, respectively. The clinical failure rates for patients aged below 40 and those aged 40 and over were 20.5% and 39.0%, respectively (Table 2). The radiological failure rates for patients belonging to ARCO stage II, IIIa, and III (b + c) were 30.9%,35.2%, and 81.5%. The KM survival curve showed that the survival rate of patients belonging to ARCO stage III (b + c) was lower than that of patients belonging to ARCO stages IIIa and II. There were no significant differences in the survival rates of patients belonging to ARCO stages IIIa and II (Figure 2). The survival rates of patients with L2 and L3-type disease were lower than those of patients with (C + M + L1)-type disease. There were no significant differences in the survival rates of patients with L2 and L3 type disease (Figure 1). |
|  |  | (*b*) Report category boundaries when continuous variables were categorized |  | table 2 |
|  |  | (*c*) If relevant, consider translating estimates of relative risk into absolute risk for a meaningful time period |  |  |

Continued on next page

| Other analyses | 17 | Report other analyses done—eg analyses of subgroups and interactions, and sensitivity analyses | 5 |  |
| --- | --- | --- | --- | --- |
| Discussion | | | | |
| Key results | 18 | Summarise key results with reference to study objectives | 8 | In summary, disease type, stage, and patient age are risk factors that impact surgical failure rates. Bone grafting through a window at the femoral head-neck junction performed in patients with no femoral head collapse or in those with a degree of collapse <2 mm (ARCO stage IIIa) showed good clinical success rate. Patients with a degree of femoral head collapse >2 mm (ARCO stage IIIb and IIIc) and those with necrotic lesions involving the lateral pillar (L2 and L3 type) had a high rate of surgical failure. Further, patients aged 40 and over had worse post-operation prognosis than patients aged below 40. |
| Limitations | 19 | Discuss limitations of the study, taking into account sources of potential bias or imprecision. Discuss both direction and magnitude of any potential bias |  | There are some limitations in this study. This study is limited by virtue of the retrospective analysis of only one center. And there was no randomized and blinded control group with conservative in this study. |
| Interpretation | 20 | Give a cautious overall interpretation of results considering objectives, limitations, multiplicity of analyses, results from similar studies, and other relevant evidence | 6-7 |  |
| Generalisability | 21 | Discuss the generalisability (external validity) of the study results | 6-7 |  |
| Other information | |  | | |
| Funding | 22 | Give the source of funding and the role of the funders for the present study and, if applicable, for the original study on which the present article is based |  | National Natural Science Foundation of China(81372013) |

*Give information separately for cases and controls in case-control studies and, if applicable, for exposed and unexposed groups in cohort and cross-sectional studies.

**Note:** An Explanation and Elaboration article discusses each checklist item and gives methodological background and published examples of transparent reporting. The STROBE checklist is best used in conjunction with this article (freely available on the Web sites of PLoS Medicine at http://www.plosmedicine.org/, Annals of Internal Medicine at http://www.annals.org/, and Epidemiology at http://www.epidem.com/). Information on the STROBE Initiative is available at www.strobe-statement.org.
